# Gaseous Products Evolution Analyses for Catalytic Decomposition of AP by Graphene-Based Additives

**DOI:** 10.3390/nano9050801

**Published:** 2019-05-24

**Authors:** Shuwen Chen, Ting An, Yi Gao, Jie-Yao Lyu, De-Yun Tang, Xue-Xue Zhang, Fengqi Zhao, Qi-Long Yan

**Affiliations:** 1 Science and Technology on Combustion, Internal Flow and Thermostructure Laboratory, Northwestern Polytechnical University, Xi’an 710072, China; shuwenchen@nwpu.edu.cn (S.C.); jieyaolv@mail.nwpu.edu.cn (J.-Y.L.); tangdy@mail.nwpu.edu.cn (D.-Y.T.); xuexuezhang@mail.nwpu.edu.cn (X.-X.Z.); 2 Science and Technology on Combustion and Explosion Laboratory, Xi’an Modern Chemistry Research Institute, Xi’an 710065, China; anting715@163.com; 3 School of Astronautics, Northwestern Polytechnical University, Xi’an 710072, China; gy0704@163.com

**Keywords:** thermolysis, energetic materials, GO-based catalysts, quantitative analyses, decomposition mechanisms

## Abstract

A quantitative evaluation method has been developed to study the effects of nanoadditives on thermal decomposition mechanisms of energetic compounds using the conventional thermogravimetry coupled with mass spectrometry (TG/MS) technique. The decomposition of ammonium perchlorate (AP) under the effect of several energetic catalysts has been investigated as a demonstration. In particular, these catalysts are transition metal (Cu^2+^, Co^2+^ and Ni^2+^) complexes of triaminoguanidine (TAG), using graphene oxide (GO) as dopant. They have been well-compared in terms of their catalytic effects on the concentration of the released gaseous products of AP. These detailed quantitative analyses of the gaseous products of AP provide a proof that the proton transfer between ∙O and O_2_ determines the catalytic decomposition pathways, which largely depend on the type of reactive centers of the catalysts. This quantitative method could be applied to evaluate the catalytic effects of any other additives on the thermal decomposition of various energetic compounds.

## 1. Introduction

Energetic materials (EMs) are widely used as propellants, explosives, and pyrotechnics. The conventional EMs, such as 1,3,5-trinitro-1,3,5-triazinane (RDX), 1,3,5,7-tetranitro-1,3,5,7-tetrazocane (HMX), and ammonium perchlorate (AP), are still playing dominant roles in the formulations. The nano-sized additives are usually used to improve their performances and more innovative additives have been designed and used during past decades [1,2,3]. The decomposition and combustion of EMs are the key parameters that have to be investigated before their applications, which are strongly connected with their compatibility, safety, and performance. The decomposition has been found to be the initial stage of combustion, and it should be well-evaluated at laboratory scale. AP is a well-known oxidizer in solid composite propellants. In order to get the burning behavior of propellants, researchers usually focus on their thermal property first [4,5]. The thermal behavior of AP has been widely studied during the past several decades [6,7]. Nanoadditives have high concentrations of dislocations and large surface areas, and therefore they normally show significant catalytic effects on the decomposition of AP [8,9,10,11]. However, the nanoadditives used today in formulations are mostly inert metal oxides or metal oxide composites. To some extent, they may reduce the energy content of the propellants.

In order to overcome the limitations mentioned above, one has to develop energetic metal complexes or metal organic frameworks (MOFs) with great thermal stability and compatibility as energetic catalysts. It has been recently shown that graphene oxide (GO) doped transition metal complexes are promising energetic catalysts [12,13]. It has been reported that the intrinsic exothermicity of GO (1600 J g^−1^) is comparable to several hazardous chemicals and explosives [14]. In addition, GO could be considered as a stabilizing agent [15]. In our recent study, GO-doped transition metal (nickel, cobalt and copper) complexes of triaminoguanidine (TAG) were prepared, and their effects on the thermal properties and decomposition mechanisms of RDX studied [16]. These materials could not only catalyze the decomposition of RDX, but also improve the thermal stability of RDX due to their enhanced thermal conductivity. The decomposition behavior of GO complexes modified AP have been briefly studied based on conventional thermogravimetry coupled with mass spectrometry (DSC/TG) analysis [17]. The results showed that the hybrid catalysts can enhance the initial decomposition temperature and change the thermolysis mechanism by introducing different types of metal ions. But the detailed effect of nanoadditives, especially for GO-based energetic catalysts, on thermolysis products and reaction pathways of AP are not well investigated [18]. According to the literature, two mechanisms have been proposed for the thermal decomposition of AP: (a) electron transfer mechanism [19] and (b) proton transfer mechanism [20], depending on the temperature. A large amount of data has shown that the GO-TAG based catalysts have significant effects on heat releases and thermal stability of EMs, but the inherent chemical mechanisms are still not well-known.

Therefore, this paper intends to present a comprehensive quantitative analysis to clarify how nanoadditives affect the decomposition mechanisms of EMs. The catalytic effects of GO-based energetic additives on thermal decomposition chemical pathways and gaseous products of AP have been investigated as a typical demonstration. It is a novel method to get the catalyst mechanism by measuring the amount of gaseous decomposition products. Thermal analysis techniques including thermogravimetry coupled with mass spectrometry (TG/MS) were employed to evaluate the decomposition mechanisms [21,22,23]. This technique was used to evaluate the bond breaking and gaseous products formation during thermal decomposition of EMs. Thus, this work aims at studying the thermal decomposition of AP, in presence of various GO-TAG based energetic catalysts, on the basis of TG/MS technique.

## 2. Experimental Procedure

### 2.1. Sample Preparation

G-T-M and TAG-M composites were synthesized followed by the method reported in the previously published paper ([16], the preparation was summarized in the supporting materials). In order to get AP contained complex, a saturated solution of AP containing 160 mg of AP and 20 mL of acetone was prepared first. Then 40 mg TAG-M or G-T-M was added to this solution (M means Nickel, Cobalt or Copper ion), and stirred for 3 h at room temperature. The final G-T-M/AP and TAG-M/AP complexes were obtained after freeze drying. Warning—the solvent-free TAG-Cu complex would undergo self-ignition and fast deflagration to detonation transition reaction easily in the presence of oxygen.

G-T-M indicates GO-doped transition metals complexes of TAG. TAG-M is transition metals modified TAG. G-T-M/AP and TAG-M/AP correspond to G-T-M and TAG-M doping AP, respectively. The components of G-T-M/AP and TAG-M/AP have been summarized in Table 1.

### 2.2. Experimental Techniques

The gaseous products from thermal decomposition of AP and AP-based mixtures using TAG-M and G-T-M as energetic nanoadditives were detected using the TG-DSC/MS technique. This experiment was realized on a simultaneous thermoanalyzer STA 449 F3 coupled with a quadrupole mass spectrometer QMS 403 C Aëolos (Netzsch Group, Selb, Germany). An alumina pan with a pin-hole cover was used as a sample pan). The sample masses for these measurements were about 3 mg, with a heating rate of 10 °C/min in a temperature range of 40–500 °C, using an argon atmosphere (gas flow: 50 mL/min).

## 3. Results and Discussion

### 3.1. Possible Catalytic Decomposition Pathways of AP

According to the literature [24,25,26], there are two possible decomposition processes for AP; the low and high temperature stages. Since AP contains plenty of nuclei with tiny pores [27], they are likely to provide highly active sites on their surface at the low temperature decomposition stage, while the high-temperature decomposition stage takes place at the surfaces of the nanocrystals, and involves adsorption and desorption of ammonia as well as perchloric acid. The decomposition of AP generally follows two steps [28,29]; a solid-gas multiphase reaction of the first decomposition step at 300–330 °C, and a gas phase reaction of the second decomposition step at 450–480 °C. The possible decomposition reactions and transformations are as follows [27]:

NH_4_ClO_4_ → NH_3_ + HClO_4_

HClO_4_ → 2O_2_ + HCl

O_2_ → O_2_^−^

4NH_4_^+^ + O_2_^−^ → 2H_2_O + 4NH_3_

7ClO_4_^−^ → 2ClO_3_ + ClO_2_ + 2ClO + 9O_2_ + Cl_2_

4NH_3_ + 5O_2_ → N_2_O + NO + NO_2_ + 6H_2_O

As mentioned above, the TG/MS technique has been used to detect the products of thermal decomposition of AP and TAG-based catalysts coated AP. The major gas products have been found to be O_2_, NO, NH_3_, H_2_O, ∙O, which would be used to clarify the possible catalytic decomposition mechanisms.

**Figure 1:**
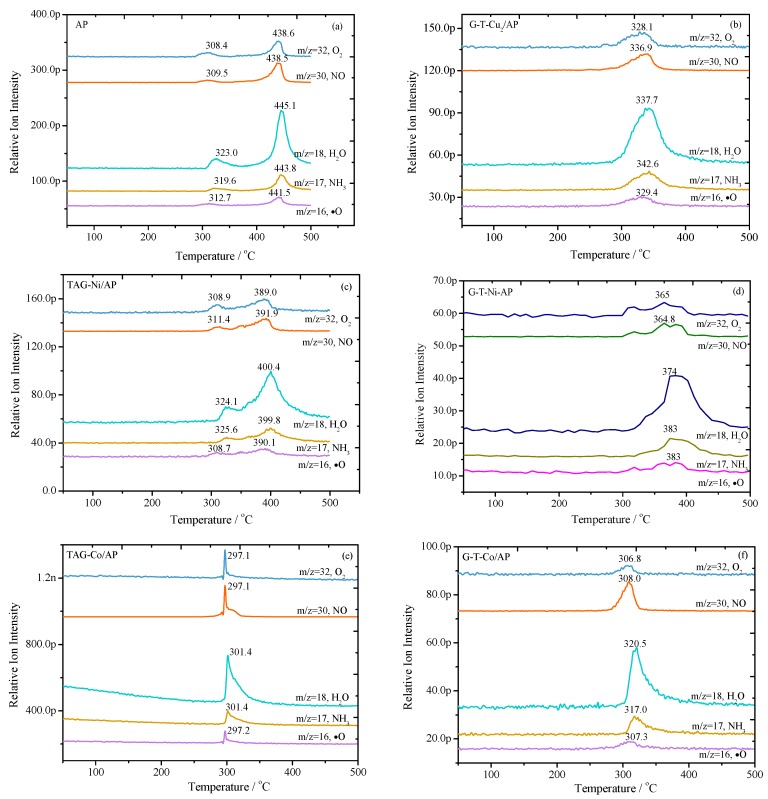
Temperature dependency of the ion flows from non-isothermal decomposition for pure AP and coated AP under a heating rate of 10 °C min^−1^ using thermogravimetry coupled with mass spectrometry (TG/MS) technique: (**a**) pristine AP; (**b**) G-T-Cu_2_/AP; (**c**) TAG-Ni/AP; (**d**) G-T-Ni/AP; (**e**) TAG-Co/AP; (**f**) G-T-Co/AP; TAG—triaminoguanidine.

As shown in Figure 1a, there are two obvious gas evolution peaks at around 308–323 °C and 438–445 °C for pure AP, which are attributed to the low-temperature decomposition and high-temperature decomposition, respectively. Compared with pure AP, G-T-Cu_2_/AP shows only one gas release peak between 328 °C and 342 °C. The G-T-Cu_2_/AP decomposes with a single but wide releasing peak, so G-T-Cu_2_ has a catalyst effect on AP decomposition. In the presence of TAG-Ni, the decomposition peak of the first step has the same temperature range as that of pure AP, and the second peak temperature has decreased by about 40 °C. The addition of TAG-Ni has a larger effect on the releasing amount of NO and ∙O. For the mixture of G-T-Ni/AP, as shown in Figure 1d, there is only one decomposition peak similar to that of G-T-Cu_2_/AP, but with a higher peak temperature in the range of 364–383 °C. The results suggest that G-T-Ni can improve the thermal stability of AP compared with G-T-Cu_2_.

A sharp decomposition peak can be observed for TAG-Co/AP with the range of 297–301 °C, so the presence of TAG-Co has a large catalytic effect on AP decomposition by decreasing the amount of released gases and decomposition temperature. All the gas products are releasing at almost the same temperature. Regarding G-T-Co/AP, the decomposition peak is smoother and the peak temperature increases by 10–20 °C with the addition of GO. It has been reported that G-T-Co can slightly increase the thermal stability of AP [17]. The results indicate that TAG-M complexes in the presence of GO can enhance thermal stability for AP, resulting in slower reaction rates at a higher temperature range.

### 3.2. The Dependence of Gases’ Evolution Processes on Temperature for Catalytic Decomposition of AP

**Figure 2:**
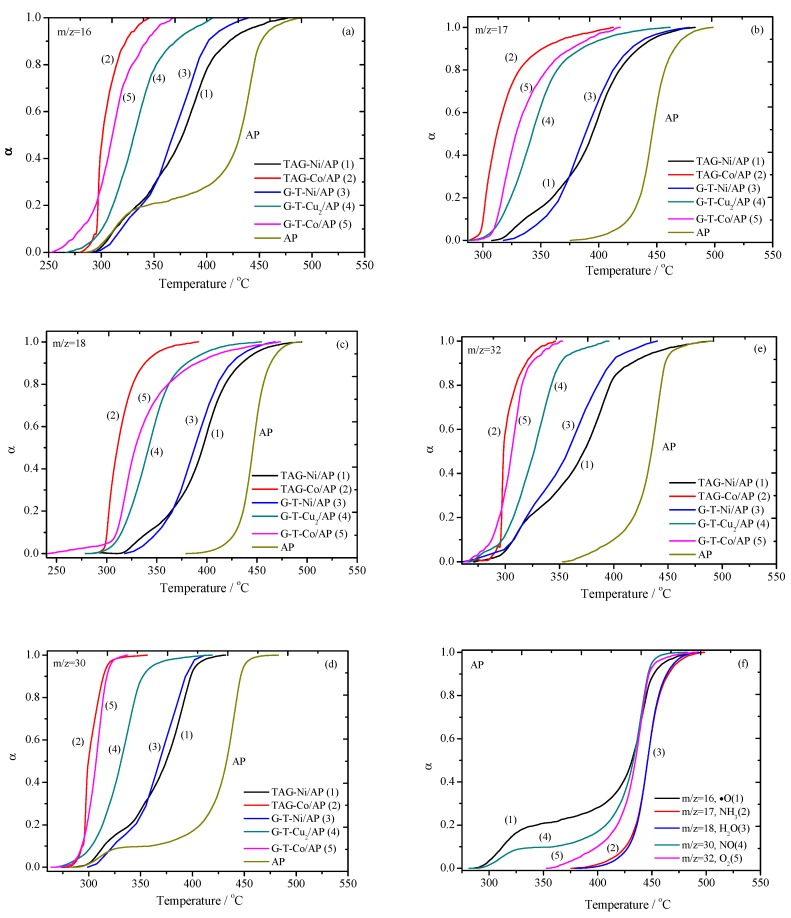
The dependence of accumulation (α is conversion rate) of the typical gaseous products on the temperature for AP-based mixtures: (**a**) *m/z* = 16, ∙O; (**b**) *m/z* = 17, NH_3_; (**c**) *m/z* = 18, H_2_O; (**d**) *m/z* = O_2_; (**e**) *m/z* = 30, NO; (**f**) summary of products evolution processes of pristine AP.

The curves in Figure 1 were derived and summarized as a function of gaseous product types, as shown in Figure 2.

For the decomposition of pure AP (Figure 2f), the sequence of its gas releasing process is as follows: NO, ∙O, O_2_, NH_3_ and H_2_O. The releasing rate of NO and ∙O increases rapidly at around 300 °C, and then slows down when the temperature reaches 350 °C. In the meantime, the other three kinds of gases start to release. The releasing rates of all the gaseous products reach their maximum values at the temperature of 420 °C, and start to decrease when the temperature is over 450 °C. The two releasing stages correspond to two decomposition peaks in Figure 1a. Compared with pure AP, the addition of TAG-M complexes could affect the gaseous releasing processes due to the change of chemical reaction pathways, which will be discussed in more detail in a later section.

There is only a trace amount of ∙O (*m/z* = 16) radical being formed for all involved samples. The release of ∙O is even earlier than that of pure AP in the presence of G-T-Co, G-T-Cu_2_ and TAG-Co complexes, but TAG-Ni and G-T-Ni complexes postponed the reactions that would release ∙O. The mass loss of AP in these cases should be largely caused by the transformation of O_2_→O_2_^−^ that forms ∙O radical, which has been demonstrated by the curve of *m/z* = 16 in Figure 1. Five kinds of TAG-based complexes have similar catalytic effects on reaction temperature, which lead to the formation of NH_3_ (*m/z* = 17) and H_2_O (*m/z* = 18), where the releasing rate of gas is increased. In comparison, G-T-Ni postpones the temperature at which NO (*m/z* = 30) starts to release. It also improves the thermal stability of AP, whereas the other four types of G-T-M complexes decrease the initial gas releasing temperature of AP. The initial decomposition temperature of AP is decreased, and the reaction rate is increased in formation of O_2_ (*m/z* = 32), by using TAG-M complexes as additives.

In the presence of TAG-Ni, all the gas release processes occur in two steps, which is similar to the case of pure AP. G-T-Ni/AP has a lower initial temperature for releasing O_2_ and ∙O than that of TAG-Ni/AP. However, the initial releasing temperature for NO, NH_3_ and H_2_O of G-T-Ni/AP is higher than that of TAG-Ni/AP. If comparing G-T-Co/AP with TAG-Co/AP, the initial temperatures for the production of NO, O_2_, ∙O and H_2_O are much lower, where extra NH_3_ was generated. The gas releasing rate of TAG-Co/AP is also higher than that of G-T-Co/AP throughout their decomposition. The G-T-Cu_2_ has a significant catalytic effect on AP decomposition so that the stabilization of GO is excluded. It is clear that the initial temperature of G-T-Cu_2_ has been decreased in comparison to pure AP. In summary, the addition of TAG-M complexes to AP may lead to significant changes in the chemical decomposition pathways.

### 3.3. Quantitative Analyses of Gaseous Products’ Changes of AP in Presence of These Nanocatalysts

To study the catalytic effect of TAG-based complexes on the decomposition mechanisms of AP, the ion flow curves (Figure 1) have been integrated and analyzed. In order to make a quantitative comparison, the characteristic parameters of these curves are calculated and summarized in Table 2.

Many investigations have shown that AP decomposition proceeds via the electron transfer from the cation NH_4_^+^ to anion ClO_4_^−^, and the catalytic processes with nanocrystalline additives involve the electron transfer between AP and nanoadditives. Boldyrev [28] concluded that the AP decomposition process is followed by a proton transfer from the cation NH_4_^+^ to anion ClO_4_^−^. We may assume that the addition of TAG-M complex has catalytic activity in AP decomposition. The amount of gaseous product is different depending on the type of catalysts. For TAG-M doped by GO, the nanocrystalline of TAG-M grows uniformly on the graphene nanosheets, which can increase the contact surface area between AP with the active electron transfer centers, thus resulting in accelerated thermolysis reaction processes. The GO-doped TAG-M complexes with different metal centers could either postpone or accelerate the initial decomposition of AP. According to the quantitative change of gases by TG-DSC/MS technique, the reaction pathways have been greatly changed by these catalysts, depending on the type of metal ions.

For releasing ∙O, TAG-Ni and G-T-Ni have little influence on the initial temperature of decomposition, but G-T-Ni/AP can help increase the reaction rate compared with TAG-Ni/AP and AP. The addition of TAG-Co, G-T-Co or G-T-Cu_2_ increases the initialization temperature and shortens the reaction time. For the reaction to produce NH_3_, TAG-M complex decreased the initial reaction temperature of NH_3_ by 57.6–88.4 °C while increasing the reaction time. For the reaction to produce NO, G-T-Ni/AP postpones the initial reaction temperature, while the other nano- complexes decrease the initialization temperature. All of these TAG-M complexes have increased the reaction rate.

TAG-Co has shortened the reaction that produces H_2_O, whereas the other four types of complexes have prolonged the reaction time by 53.8% to 100%. The initial decomposition temperature of AP under the effects of all TAG-M complexes have been decreased by 61 °C to 138 °C. For the releasing of O_2_, TAG-Co based complex has shortened the reaction time by 35–40%, but Ni-based and Cu-based composites prolong the reaction time by 29–57%. Cu-based component reduces the initial decomposition time and slightly decreases the reaction time by 5.8%.

**Figure 3:**
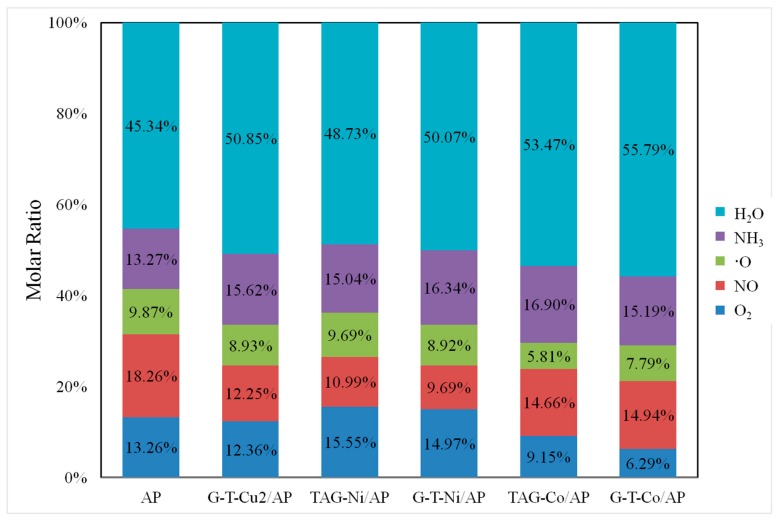
Comparison of molar ratio of gas products decomposed by AP coated with different TAG-M based catalysts.

The amount of each gas product is quantified by integrating the peak area of ion intensity curves in Figure 1. The percentage of major gaseous products for each TAG-M complex was calculated and summarized in Figure 3. The amount of H_2_O and NH_3_ was increased with the addition of G-T-Cu_2_, while the amount of the other three kinds of gases was reduced. This suggests that G-T-Cu benefits the reaction of NH_4_^+^ and ClO_4_^−^, but suppresses the reaction of oxygen conversion. For TAG-Ni/AP and G-T-Ni/AP, the reactions of producing NO and ∙O were suppressed, but the reactions producing H_2_O, NH_3_ and O_2_ were promoted. The amount of change of gas released for G-T-Ni/AP is more significant than that of TAG-Ni/AP. For TAG-Ni/AP, the molar ratio of ∙O and H_2_O changed by −1.8% and 7.5%, respectively. Additionally, TAG-Ni/AP shows two decomposition peaks the same as that of pure AP. The TAG-Co based additives show a similar catalyst effect to that of TAG-Cu_2_ complex. The reaction of ∙O is very sensitive to TAG-Co, which reduces the gas amount by 40%. The decomposition curve of TAG-Co/AP is the sharpest and its reaction time is the shortest. G-T-Co/AP shows the greatest influence on the reaction of producing O_2_, with a decrease in amount of gas by 52%.

The addition of TAG-M complex has an obvious catalytic effect on the whole decomposition efficiency of AP, where an increase of gas production of H_2_O and NH_3_ could be found. For the molar ratio of ∙O, TAG-Ni/AP presents the lowest reduction rate of gas production at 1.8%, compared to 41.1% for that of TAG-Co/AP. The amount of O_2_ generation was reduced for TAG-Co/AP, G-T-Co/AP, and G-T-Cu_2_/AP, while it increased in TAG-Ni/AP and G-T-Ni/AP. Co-based complex shows an obvious reduction in O_2_ product, among which G-T-Co/AP presents a reduction rate as high as 52.5%. The producing of NO is the most sensitive to G-T-Ni, with a reduction rate of 46.9%.

**Figure 4:**
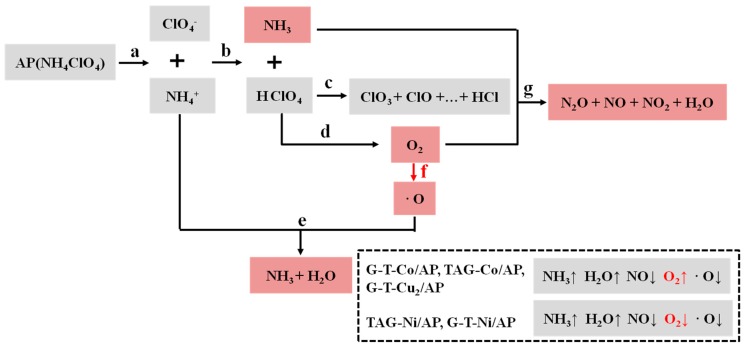
Thermal decomposition mechanisms of AP under the catalytic effects of TAG-M complexes (arrow up means increase and arrow down means decrease).

Figure 4 shows that there is a pair of ions (NH_4_^+^ and ClO_4_^−^) in NH_4_ClO_4_ crystal lattice. The proton transfers from NH_4_^+^ to ClO_4_^−^, which leads to reaction *b* and the formation of NH_3_ and HClO_4_. Moreover, the graphene could be combined with metal oxides to improve the catalytic activity. For instance, the molar ratio change of G-T-Cu_2_, TAG-Co, and G-T-Co show the same trend; the amount of H_2_O and NH_3_ is increasing, while the amount of ∙O, NO and O_2_ is decreasing. According to the amount of change, we can postulate that reactions *a*, *f*, and *e* were promoted (Figure 4).

For TAG-Co catalyst dispersed in AP, the amount of ∙O has the minimum value, where one sharp decomposition peak was observed from the ion intensity curve. In the case of G-T-Co/AP, the amount of O_2_ shows the maximum reduction, while graphene shows additional promotion to reaction *f*. In contrast, TAG-Ni catalyst promotes reaction *d*, but suppresses reaction *g*, while the amount of ∙O slightly decreases compared with that of pure AP. G-T-Ni shows catalytic effect on reaction *d*, but suppresses reactions *f* and *g*.

All TAG-M catalysts could promote the reactions of AP decomposition by increasing the amount of gas products. In addition G-T-Co, TAG-Co, and G-T-Cu_2_ have stronger catalytic effects than nickel-based compounds, as they could make two-step gas releasing into one step. This conclusion is consistent with the thermal results from our previous research [17], which claimed that all TAG-M based composites could catalyze the first decomposition process of AP, and Co-based and Cu-based materials have stronger catalytic effects.

## 4. Conclusions

TG/MS was used to quantitatively study the effects of additives on decomposition mechanisms of energetic compounds, where catalytic decomposition of AP by GO-based catalysts has been selected as a typical example. It has been demonstrated that the detailed quantitative analyses of the gaseous products of AP would show the inherent mechanism changes under the effects of various additives. This method could be applied to analyze the decomposition mechanisms of any other energetic compounds.

The findings further support the literature on the catalytic decomposition kinetics of AP. The GO-based catalysts show improved catalytic efficiency due to their capability in increasing the conversion rates of NH_3_ and H_2_O. This can be explained by more O elements being transferred to react with NH_4_^+^, which enhances the initial decomposition heat, resulting in the combination of two decomposition peaks. The amount of O_2_ decreases under the effect of Ni-based complexes, which promote the reaction in producing ∙O. In particular, new findings suggest that TAG-Ni and G-T-Ni materials have higher stabilization effects on AP than the others. The Co-based and Cu-based composites could increase the release of O_2_, so they have better catalytic effect on AP decomposition. The improved reactions between ∙O and the other radicals mean a better catalytic effect on the decomposition of AP.

## Figures and Tables

**Table 1:** The compositions of ammonium perchlorate (AP)-based mixtures (in weight percent).

	AP	GO	TAG	Metal
G-T-Co/AP	80%	0.4%	5.0%	14.6% Cobalt
G-T-Cu_2_/AP	80%	0.4%	5.7%	13.9% Copper
G-T-Ni/AP	80%	0.5%	6.6%	12.9% Nickel
TAG-Co/AP	80%	-	6.3%	13.7% Cobalt
TAG-Ni/AP	80%	-	7.1%	12.9% Nickel

**Table 2:** A summary of TG/MS parameters of AP coated by different GO (graphene-oxide)-based catalysts.

*m/z* = 16, ∙O					
	*T*_o_	*T*_e_	∆*T*	n	n_m_
TAG-Ni/AP	289.9	478.0	188.1	4.541 × 10^−10^	9.69%
TAG-Co/AP	279.4	346.7	67.3	5.803 × 10^−10^	5.81%
G-T-Ni/AP	289	440	151	1.969 × 10^−10^	8.92%
G-T-Cu_2_/AP	266.2	406.7	140.5	3.653 × 10^−10^	8.93%
G-T-Co/AP	251.5	369.2	117.7	1.330 × 10^−10^	7.79%
AP	282.7	491.2	208.5	5.734 × 10^−10^	9.87%
***m/z* = 17, NH_3_**					
TAG-Ni/AP	307.8	483.1	175.3	7.052 × 10^−10^	15.04%
TAG-Co/AP	287.6	412.5	124.9	16.89 × 10^−10^	16.90%
G-T-Ni/AP	318	478	160	3.607 × 10^−10^	16.34%
G-T-Cu_2_/AP	287.2	461.5	174.3	6.389 × 10^−10^	15.62%
G-T-Co/AP	291.2	418.4	127.2	2.595 × 10^−10^	15.19%
AP	375.6	498.4	122.8	7.706 × 10^−10^	13.27%
***m/z* = 18, H_2_O**					
TAG-Ni/AP	292.5	494.6	202.1	22.849 × 10^−10^	48.73%
TAG-Co/AP	282.3	391.8	109.5	53.426 × 10^−10^	53.47%
G-T-Ni/AP	318	468	150	11.052 × 10^−10^	50.07%
G-T-Cu_2_/AP	278.7	454.5	175.8	20.80 × 10^−10^	50.85%
G-T-Co/AP	241.3	473.3	232	9.531 × 10^−10^	55.79%
AP	379.2	493.5	114.3	26.328 × 10^−10^	45.34%
***m/z* = 30, NO**					
TAG-Ni/AP	273.7	431.9	158.2	5.153 × 10^−10^	10.99%
TAG-Co/AP	277.7	356.5	78.8	14.65 × 10^−10^	14.66%
G-T-Ni/AP	299	412	113	2.138 × 10^−10^	9.69%
G-T-Cu_2_/AP	265.3	419.0	153.7	5.010 × 10^−10^	12.25%
G-T-Co/AP	263.7	337.6	73.9	2.552 × 10^−10^	14.94%
AP	281.5	482.8	201.3	10.604 × 10^−10^	18.26%
***m/z* = 32, O_2_**					
TAG-Ni/AP	270.8	488.9	218.1	7.291 × 10^−10^	15.55%
TAG-Co/AP	266.4	346.3	79.9	9.146 × 10^−10^	9.15%
G-T-Ni/AP	261	440	179	3.305 × 10^−10^	14.97%
G-T-Cu_2_/AP	265.0	395.5	130.5	5.055 × 10^−10^	12.36%
G-T-Co/AP	262.9	352.6	89.7	1.075 × 10^−10^	6.29%
AP	352.8	491.4	138.6	7.701 × 10^−10^	13.26%

Notes: *T*_o_, onset temperature of decomposition, in °C; *T*_e_, the end temperature of decomposition, in °C; ∆*T*, *T*_e_ − *T*_o_, in °C; n, the amount of each released gas; n_m_, proportion of each product in total gaseous products.

## Supplementary Materials

The following are available online at https://www.mdpi.com/2079-4991/9/5/801/s1, Figure S1: The G-T-M (M=Cu^2+^, Ni^2+^ and Co^2+^) coordination nanomaterials prepared by reaction of ammonized GO with corresponding metal nitrates: mononuclear coordination complexes could be formed based on triaminoguanidine ligand, Figure S2: The SEM photos of involved materials, where G-T-Cu, G-T-Co, G-T-Ni, TAG-Co and TAG-Ni are presented.
