# Supplementary material for: Gaseous Products Evolution Analyses for Catalytic Decomposition of AP by Graphene-Based Additives
Source: Nanomaterials (Basel). 2019 May 24;9(5):801. doi: 10.3390/nano9050801 (PMC6587332; doi:10.3390/nano9050801)
Supplement: Supplementary file 1 [file nanomaterials-09-00801-s001.pdf]

Supporting Information

Gaseous Products Evolution Analyses for Catalytic Decomposition of AP by Graphene-Based Additives

Shuwen Chen [1], Ting An [2], Yi Gao [3], Jie-Yao Lyu [1], De-Yun Tang [1], Xue-Xue Zhang [1], Fengqi Zhao [2,*] and Qi-Long Yan [1,*]

[1] Science and Technology on Combustion, Internal Flow and Thermostructure Laboratory, Northwestern Polytechnical University, Xi'an 710072, China; shuwenchen@nwpu.edu.cn (S.C.); jieyaolv@mail.nwpu.edu.cn (J.-Y.L.); tangdy@mail.nwpu.edu.cn (D.-Y.T.); xuexuezhang@mail.nwpu.edu.cn (X.-X.Z.)

[2] Science and Technology on Combustion and Explosion Laboratory, Xi'an Modern Chemistry Research Institute, Xi'an 710065, China; anting715@163.com

[3] School of Astronautics, Northwestern Polytechnical University, Xi'an 710072, China; gy0704@163.com

* Correspondence: qilongyan@nwpu.edu.cn (Q.-L.Y.); zhaofqi@163.com (F.Z.)

Received: 13 March 2019; Accepted: 17 May 2019; Published: 24 May 2019

1. Preparation of TAG-M and G-T-M

The detailed preparation method can be found in our recently published paper [16]. For the preparation of TAG-M, 2.4 mmol of TAG·HNO_3 were dissolved into 40 mL distilled water, then 3 mmol of cobalt nitrate hexahydrate, copper nitrate trihydrate, and nickel nitrate hexahydrate were added into the solution above, respectively. After the reaction at 75 °C for 2 h, $NaHCO_3$ was added to neutralize the acid. Lastly, the precipitates were filtrated and washed by ethanol. (Caution: TAG-M complexes without solvent are sensitive and can explode in the air.)

The preparation of G-T-M: 200 mg GO was dispersed in 200 mL distilled water by ultrasonication, then heated to 70 °C. 50 mL of 1-ethyl-3-(3-(dimethylamino)propyl) carbodiimide (EDC) and 30 mg of N-hydroxysuccinimide (NHS) were added to GO suspension and stirred for 30 min. Then 2500 mg of triaminoguanidine hydrochloride (TAG·HNO_3) was added to the obtained mixture, which should be kept in 70 °C for 4 h while stirring. The black flocculent precipitates were gradually formed, which were filtered and washed by distilled water. The 960 mg of the above mixtures were divided into three parts, and 3 mmol of cobalt nitrate hexahydrate, trihydrate nitric acid copper, and nickel nitrate hexahydrate were added to them, respectively. These mixtures have reactions at 75 °C for 2 h. Then a certain amount of $NaHCO_3$ was added to neutralize the acid. Finally, the precipitates were filtrated and washed by ethanol. The molecular structures of abovementioned G-T-M are shown in Figure S1.

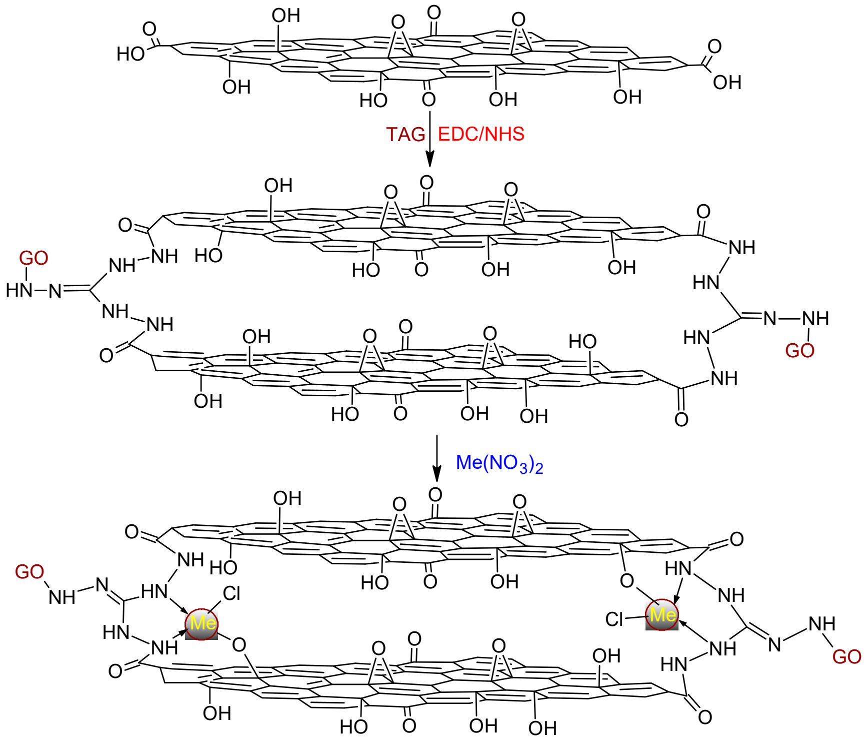

Figure S1. The G-T-M (M = Cu^{2+}, Ni^{2+} and Co^{2+}) coordination nanomaterials prepared by the reaction of ammonized GO with corresponding metal nitrates; mononuclear coordination complexes could be formed based on triaminoguanidine ligand.

2. Characterizations and Performances

The SEM analysis was performed on a ZEISS ΣIGMA with a working distance of 5 mm at 5 kV or 10 kV accelerating voltage.

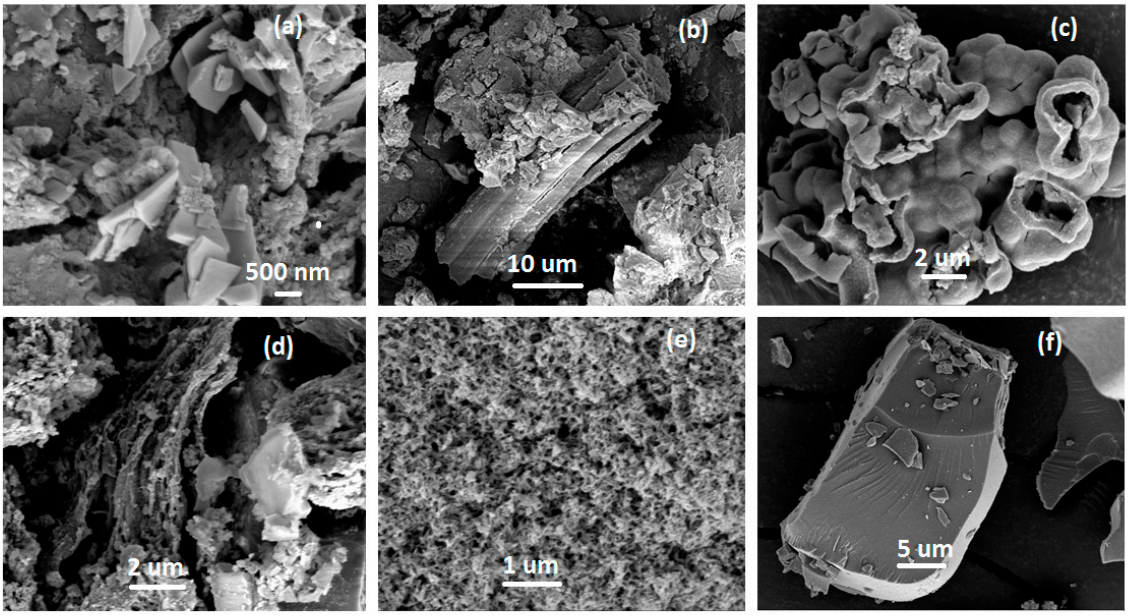

Figure S2. The SEM photos of involved materials, where G-T-Cu (**a**), G-T-Co (**b**,**c**), G-T-Ni (**d**), TAG-Co (**e**) and TAG-Ni (**f**) are presented.

References

1. [s1] Yan, Q.-L.; Petrutik, N.; Shlomovich, A.; Burstein, L.; Pang, S.-P.; Gozin, M. Highly insensitive and thermostable energetic coordination nanomaterials based on functionalized graphene oxides, *J. Mater. Chem. A*. **2016**, *108(10)*, 111–117.
2. [s2] Chen, S.; He, W.; Luo, C.-J.; An, T.; Chen, J.; Yang, Y.; Liu, P.-J.; Yan, Q.-L. Thermal behavior of graphene oxide and its stabilization effects on transition metal complexes of triaminoguanidine, *J. Hazard. Mater.*, **2019**, *368*, 404 - 411.
